# Development of a Financial Toxicity Screening Tool for Radiation Oncology: A Secondary Analysis of a Pilot Prospective Patient-Reported Outcomes Study

**DOI:** 10.1016/j.adro.2021.100782

**Published:** 2021-09-11

**Authors:** Rahul N. Prasad, Tejash T. Patel, Scott W. Keith, Harriet Eldredge-Hindy, Scot A. Fisher, Joshua D. Palmer

**Affiliations:** a The James Cancer Hospital and Solove Research Institute, The Ohio State University Wexner Medical Center, Columbus, Ohio; b Doctor of Osteopathic Medicine/Master of Business Administration Program, Philadelphia College of Osteopathic Medicine, Philadelphia, Pennsylvania; c Department of Radiation Oncology, Sidney Kimmel Cancer Center at Thomas Jefferson University, Philadelphia, Pennsylvania; d Department of Pharmacology & Experimental Therapeutics, Division of Biostatistics, Thomas Jefferson University, Philadelphia, Pennsylvania; e Department of Radiation Oncology, University of Louisville School of Medicine, Louisville, Kentucky

## Abstract

**Purpose:**

Financial toxicity is highly prevalent in oncology. Early identification of at-risk patients is essential because financial toxicity is associated with inferior outcomes. Validated general oncology screening tools are cumbersome and not specific to challenges related to radiation therapy, such as daily treatments. In the population of radiation oncology patients, no standardized, validated, rapid screening tool exists. We sought to develop a rapid, no-cost, and reliable financial-toxicity screening tool for clinical radiation oncology.

**Methods and Materials:**

We retrospectively analyzed data from a prospective survey study conducted at a large referral center with a heterogeneous population. Before treatment, a 25-item modified comprehensive survey for financial toxicity incorporating subjective and objective patient-reported measures was administered to identify factors linked to the risk of developing financial toxicity, which was defined as radiation therapy resulting in any of the following: loss of income, job, or spouse or difficulty paying for meals, housing, or transportation. We applied a logistic regression model with a stepwise, backward model selection procedure. Estimated probabilities of experiencing financial toxicity were computed using the inverse-logit transformation of the sum of patient-specific predictor values multiplied by the coefficients of the selected logistic regression model. The Youden index was used to determine a reasonable risk threshold.

**Results:**

A total of 157 patients completed the questionnaire, and 34 (22%) were assessed as experiencing financial toxicity. The model retained 3 factors: age, money owed, and copayment-related worries. It resulted in a concordance statistic of 0.85, developed with a risk threshold of 18% (Youden index, 0.59). This model conferred a sensitivity of 89%, specificity of 70%, positive predictive value of 44%, and negative predictive value of 96%.

**Conclusions:**

Our proposed financial-toxicity screen is rapid, free, sensitive, and specific, and in this study, it identified early-onset, patient-reported financial toxicity after radiation therapy with just 3 simple variables: age, money owed, and copayment-related concerns. Future research steps should include a validation cohort and identification of interventions to mitigate financial toxicity.

## Introduction

The financial implications of cancer-related therapy are a significant concern for the majority of oncology patients.^1^^,^^2^ Some studies have shown that up to 75% of patients with cancer struggle to make copayments,^3^ approximately 20% improperly take prescribed medications to defray costs,^3^ and these patients are more than twice as likely to file for bankruptcy than are patients without cancer.^4^ Direct out-of-pocket costs can be substantial, because the period from workup through therapy and surveillance can stretch from months to years and sometimes decades, with incurred copays for therapy such as radiation, surgery, and systemic therapy but also bills for office visits, procedures, imaging, and laboratory tests.^5^ However, direct costs significantly underestimate the total financial burden, because patients may encounter additional costs to manage therapy-related complications or indirect effects such as time off work, possibly leading to unemployment or homelessness.^5^ Identifying financial toxicity is essential, because it has been linked to inferior quality of life and poor health outcomes for patients with cancer.^6^, ^7^, ^8^, ^9^

A patient-reported outcome measure called the comprehensive score for financial toxicity (COST) has been previously validated in the general oncology population,^10^ but the proportion of financial issues in this population that are attributable to radiation therapy is not well established. Radiation therapy, with its daily delivery potentially over multiple weeks, poses hurdles to patients’ financial and psychosocial well-being, ranging from time off work to costs for transportation, child care, and lodging, which are distinct from the challenges faced by patients undergoing surgery or systemic therapy. To assess the risk of financial distress associated with radiation therapy, we previously conducted a prospective pilot study at a large tertiary-care center with a heterogeneous patient population and found that nearly one-fourth of radiation oncology patients reported financial toxicity attributable to their care.^11^ At that time, we also surveyed more than 200 radiation oncologists; 53% reported “significant concern” about the effect of treatment-related costs on patients and 80% citied a clinical need for a reliable screening tool for financial toxicity.^11^ Despite these concerns, to our knowledge, there is no standardized, validated, screening tool tailored to specifically predict financial distress from radiation therapy. To fill this unmet need for a simple, cost effective, reliable screening tool, we conducted a secondary analysis of our previously reported prospective survey study.

## Methods and Materials

We previously reported in extensive detail the results of a single-institution, prospective survey study using patient-reported outcome measures to characterize the prevalence of financial toxicity in a population of radiation oncology patients.^11^ Only patients receiving definitive radiation therapy were included, but patients receiving either radiation therapy alone or radiation therapy with concurrent therapy were eligible. Although a small percentage of patients were eligible for definitive-intent stereotactic body radiation therapy (SBRT), this was not routinely performed in that era at our institution, and a large majority of patients received long-course radiation therapy. Financial toxicity was defined as radiation therapy resulting in any of the following: loss of income, loss of job, loss of spouse, difficulty paying for meals, difficulty paying for rent or mortgage, and difficulty paying for transportation. Three to 6 months after completion of radiation therapy, 157 patients completed a 25-question modified COST measure questionnaire collecting key demographic and financial information and assessing their level of concern regarding the financial implications of radiation therapy.^11^ Questions were written to specifically ask about financial toxicity related to radiation therapy (eg, “Are you worried about how you will pay for your radiation treatment?”). Information collected included disease site, age, insurance type, gender, race and ethnicity, marital status, highest educational level, household income, money owed, concerns regarding the ability to pay existing bills, pay for treatment, pay the deductible, or make the copayment, and the desire to talk about costs with physicians. In the follow-up period, patients were surveyed at prespecified points to track the development of financial toxicity.

The 25-question tool was clinically onerous. To develop a streamlined, rapid screening tool, we retrospectively analyzed our initial survey results. Logistic regression was conducted to correlate the responses to survey questions with the likelihood of experiencing 1 or more forms of financial toxicity. The likelihood of developing financial toxicity based on responses to these subjective and objective variables was modeled, and a stepwise, backward model selection procedure (covariate entry parameter set to a significance level of 0.9; retention significance level, 0.15) was used to progressively retain only the most predictive variables. We chose this approach because we had no a priori understanding of which study variables would be the strongest independent predictors of financial toxicity and the 0.15 retention criterion selected no more than 3 variables, which was important because only 34 patients had financial toxicity and we did not want to overparameterize or overfit the model. In this model, estimated probabilities of experiencing financial toxicity were computed using the inverse-logit transformation of the sum of patient-specific predictor values multiplied by the coefficients of the selected logistic regression model. The Youden index was used to identify the predicted financial toxicity threshold yielding the optimal combination of model sensitivity and specificity.

## Results

Detailed descriptive statistics regarding patient demographics and other survey characteristics have been previously reported and are presented in Tables E1 and E2 in the Supplement. The heterogeneous patient population was socioeconomically diverse; patients of racial and ethnic minority populations composed nearly 30% of the total study population.^11^ Of the 157 patients who completed the survey, the majority had head and neck (22%), breast (28%), prostate (28%), and/or lung (13%) cancers. Thirty-four patients (22%) reported financial toxicity. Our modeling approach condensed the initial 25-item questionnaire down to a tool using 3 key questions evaluating age, money owed, and worries about making a copayment. Table 1 depicts the odds ratios for financial toxicity for each variable included in the selected logistic regression model, most notably age ranging from 20 to 60 years (*P* = .06); money owed ranging from $5000 to $25,000 or less (*P* < .01) or $25,000 to 45,000 (*P* = .04); and being somewhat concerned (*P* < .01) or very concerned (*P* < .01) about the copayment. This model showed excellent predictive performance with a concordance statistic of 0.85, suggesting that if 2 patients were randomly selected from the study population, 1 with and 1 without financial toxicity, the model would correctly predict a higher likelihood for financial toxicity 85% of the time for the one who developed financial toxicity.

**Table 1:** Selected logistic regression model of likelihood of experiencing financial toxicity

Factor	OR (95% CI)	*P* value
Age, y		
20-60 (n = 55) vs ≥71 (n = 32)	5.72 (0.96-34.09)	.06
61-70 (n = 69) vs ≥71 (n = 32)	2.42 (0.4-14.79)	.34
Money owed, US dollars, thousands		
5-25 (n = 32) vs blank or <5 (n = 64)	7.57 (1.93-29.76)	<.01
25-45 (n = 15) vs blank or <5 (n = 64)	5.21 (1.04-26.02)	.04
≥45 (n = 46) vs blank or <5 (n = 64)	1.98 (0.45-8.69)	.36
Worried about copay		
Somewhat (n = 39) vs no (n = 99)	6.51 (2.01-21.09)	<.01
Very (n = 16) vs no (n = 99)	20.5 (4.37-96.19)	<.01

*Abbreviation:* OR = odds ratio.

**Figure 1:**
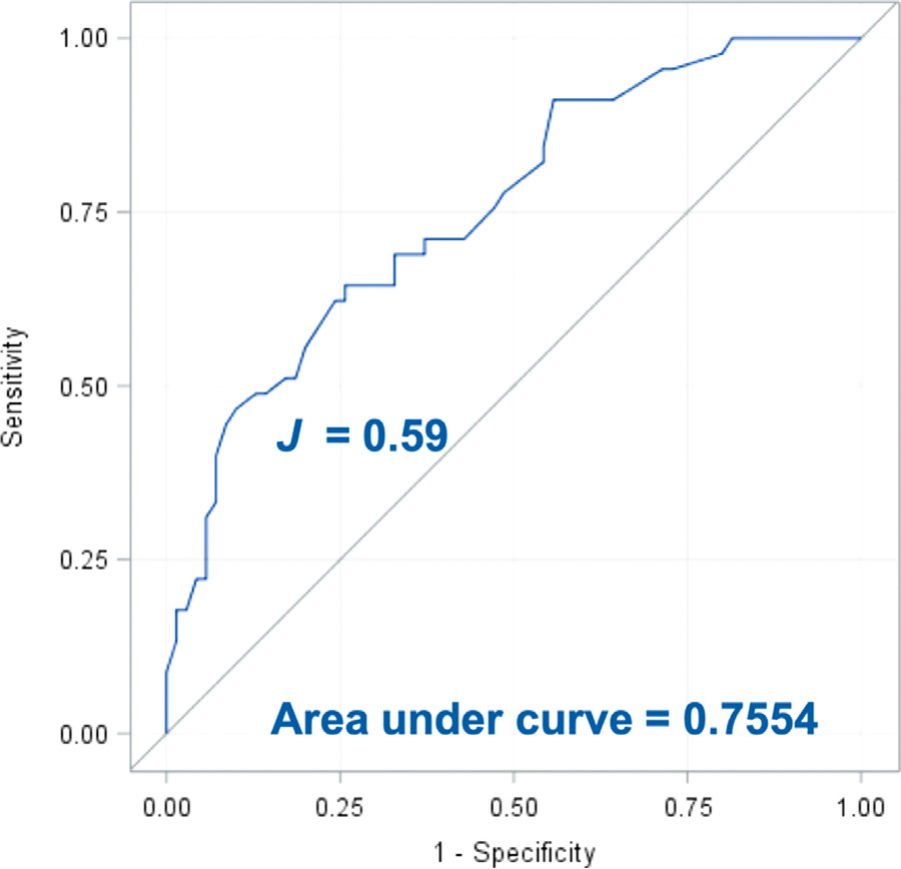
Receiver operating characteristic curve illustrating diagnostic utility of the dichotomous prediction model at various discrimination thresholds. The Youden Index (*J*) balanced sensitivity and specificity to provide a reasonable cutoff threshold.

The scoring weights for estimating patient-specific financial toxicity probabilities for each potential response for the 3 variables selected by the model are presented in Table 2. The lowest probability (1%) of financial toxicity was predicted for patients older than 70 years of age who owed less than $5000 and reported no worries about making the copayment. The highest probability of financial toxicity (91%) was estimated for patients 20 to 60 years of age who owed between $5000 and $25,000 and reported being very worried about the copayment. The receiver operating characteristic curve for the model is shown in Figure 1. For this patient population, the Youden index suggested that a model with a predicted probability of financial toxicity of 18% (Youden index, 0.59) was a reasonable choice for a financial-toxicity risk threshold because it yielded a sensitivity of 89%, specificity of 70%, a positive predictive value of 44%, and a negative predictive value of 96%. Of the 134 patients surveyed who completed the 3 questions incorporated into the model, 43 (35%) met or exceeded this risk threshold for financial toxicity.

**Table 2:** Logistic model scoring weights for computing predicted probabilities of experiencing financial toxicity

Factor	Scoring weight
Intercept	–4.447
Age, y	
20-60	1.744
61-70	0.884
≥71	0.000
Money owed, US dollars, thousands	
Blank or <5	0.000
5-25	2.024
25-45	1.650
≥45	0.684
Worried about copay	
No	0.000
Somewhat	1.873
Very	3.020

## Discussion

This retrospective analysis of a prospective, patient-reported financial toxicity study conducted at a large tertiary-care center^11^ showed the feasibility of a quick, no-cost, sensitive, and specific screening tool for patient-reported financial toxicity occurring shortly after definitive radiation therapy, relying on answers to just 3 straightforward questions. We previously found that almost 1 in 4 radiation oncology patients reported early-onset, therapy-related financial toxicity,^11^ which was consistent with the findings of general oncology meta-analyses reporting rates ranging from 28% to 48%.^1^^,^^2^ Furthermore, we asked radiation oncologists about this issue; more than half were highly concerned about patients’ ability to handle treatment-related costs, and 4 in 5 endorsed a need for a reliable screening tool in the clinic.^11^ However, there remains no standardized, validated, low-cost to no-cost, and rapid screening tool targeted to the specific needs of radiation oncology patients. Early identification of patients at risk for financial toxicity from radiation therapy is integral, because bankruptcy and other negative financial events are much more prevalent in oncology patients than in the general patient population,^3^^,^^4^ and financial toxicity has been linked to inferior quality of life and poor health outcomes.^6^, ^7^, ^8^, ^9^

The 11-question COST-measure questionnaire is currently the gold-standard, validated screening tool for financial toxicity in the oncology community.^12^ However, several potential issues limit the utility of this tool when screening radiation oncology patients. Most importantly, it was developed and validated in a population of patients with metastatic disease receiving palliative chemotherapy, which limits generalizability to radiation oncology populations receiving definitive therapy. Radiation therapy, particularly definitive, conventionally fractionated radiation therapy delivered daily for several weeks, poses different issues for patients than does surgery or systemic therapy, such as potential inability to work and transportation, lodging, and child care costs. For that reason, different socioeconomic, financial, cancer, or treatment-related variables may predict financial toxicity in radiation oncology patients, and the COST measure may have less applicability than our proposed model. Logistically, implementation of questionnaires with double-digit item counts^10^^,^^12^ is cumbersome to both patients and providers, decreasing the likelihood of completion and reliability; lengthy tools run the risk of being viewed by busy stakeholders as simply another onerous piece of paperwork.^13^ The difficulty of administrating lengthy surveys via translator to patients who do not speak English is of particular concern, because missing financial distress in this population known to be at increased risk of financial toxicity may be highly damaging.^14^ Despite these unique challenges, there are limited data regarding the prevalence of financial issues specifically attributable to radiation therapy.

Even modest concerns about making copayments featured prominently in this model that was highly predictive of financial toxicity, which is consistent with the literature suggesting that direct out-of-pocket costs are a substantial concern for patients.^3^ Additionally, mild to moderate levels of pre-existing debt were very predictive of financial toxicity in the model, whereas patients with no debt were unlikely to develop financial toxicity. This finding likely is because patients with pre-existing debt may be ill-equipped to handle additional medical costs or to survive indirect financial consequences such as time off work. For unclear reasons, owing more than $45,000 was not significantly associated with developing financial toxicity. Lastly, age was a key model component predictive for financial toxicity. Younger patients from 20 to 60 were the most likely to develop financial toxicity, which is consistent with findings from multiple retrospective studies, suggesting this effect may stem from lasting employment interruptions^14^, ^15^, ^16^ because patients with cancer tend to either partially or completely leave the workforce after therapy.^17^^,^^18^ Younger patients were also at higher risk of financial toxicity in a single-institution study screening surgical patients with gastrointestinal cancer using a 48-item screening tool.^19^ The overall rate of financial toxicity of 22% in the current study was comparable to the 34% rate seen in that surgical cohort. Psychological distress predicted financial toxicity in both the COST cohort and the current study population, which is logical given that both studies included large, socioeconomically and racially diverse populations treated at referral centers.^12^ Income was an independent factor associated with financial toxicity in both the validation cohort of the COST questionnaire and the previously mentioned surgical cohort that was not significant in this study's population.^12^^,^^19^ Additional factors predictive of economic stress in other cohorts but not in this study include race and hospital admissions in the COST validation cohort^12^ and tobacco use and lack of college education in the surgical cohort.^19^ We did not directly examine the relationship between hospital admissions and tobacco use and financial distress in this study's population. We felt that hospitalization was a less relevant risk factor for our population receiving definitive therapy, owing to earlier disease stage. Regarding income, race, and education, differences in significance between studies may relate to unexplained interactions between differences in patient stage (lower in this study than in the COST cohort) or primary treatment modality (radiation vs chemotherapy or surgery) and development of financial distress.

Notably, some patients in this study had received recent surgery or systemic therapy, and some of the captured financial toxicity events may not have resulted from radiation therapy. Although we did not collect detailed information about the delivery of systemic therapy, patients with breast or prostate cancer, composing more than half of the study population, would not have received concurrent chemotherapy. A subset of patients with head and neck or lung cancer may have received concurrent chemotherapy, but this subgroup was not large enough to allow meaningful study of the effect of combined-modality therapy on the risk of financial distress. Similarly, although we did not collect detailed data regarding treatment before radiation therapy, only the patients with breast cancer, composing less than one-third of the total study population, would generally have received radiation therapy adjuvant to surgery. The patients with prostate and lung cancers typically received definitive radiation therapy, and the paradigm for head and neck patients was mixed. Of note, the goal of this study was to calibrate a rapid screening tool to capture the financial issues faced by radiation oncology populations, but if we completely isolated the effects of radiation therapy from those of concomitant therapies, the model would underpredict financial distress in this population. Additionally, it is difficult to study a population receiving radiation therapy alone given that a large majority of malignancies are treated with multimodal therapy in concurrent or sequential fashion. Regardless, this study was unique in including a population receiving definitive radiation therapy, unlike the COST tool, which was validated in a population of patients with stage IV cancer not eligible for definitive radiation therapy.^12^ Patients receiving curative SBRT were included in this study. Although in theory, the shorter treatment course may mitigate development of treatment-related financial toxicity, copayments and other bills can still be substantial owing to the higher charges for sophisticated treatment planning. These patients still must navigate a density of appointments, even if just for a week or two, which is not seen with other therapies. Thus, we felt it was reasonable to include these patients in the study population, particularly because to our knowledge, they have not been included in previous studies.^12^ However, the model should be applied with caution to patients receiving SBRT; they composed a minor component of this study's population given the low rate of use of body SBRT at our institution in the study era, and they may face slightly different challenges than patients receiving long-course RT, owing to the condensed treatment course.

Additional limitations of this study include a patient population limited to a single, urban referral center, which may affect generalizability. However, the tertiary-care nature of the institution mitigates some of these concerns, because patients traveled for care from many distinct communities. Thus, the included patient population was socioeconomically diverse by all examined factors including household income, highest education level, insurance provider, gender, age, and marital status, with inclusion of a nearly 30% racial and ethnic minority population. Regardless, this model for patients with radiation therapy should be validated in another patient cohort because other analyses in populations receiving chemotherapy have proposed using alternate variables to predict financial distress.^12^ This study should be considered a hypothesis-generating effort to improve current modeling approaches in this patient population. Validation of the model is needed to ensure that the differences between models result from focusing on different patient populations (patients receiving definitive radiation therapy vs palliative chemotherapy) rather than features unique to this study's patient population.

Additionally, specific details regarding delivery of systemic therapy and radiation therapy were not collected up front, and owing to patient anonymization to encourage candid responses to sensitive questions, additional information could not be collected for inclusion in this analysis. Furthermore, survey bias may potentially decrease the accuracy of the model if the profile of financial toxicity in the patients who omit questionnaire items or decline the survey entirely is noticeably different from the analyzed population. Nonetheless, patient-reported outcomes are the most reliable data set in the absence of universally available, objective means to verify self-reported income, debt burden, unemployment, homelessness, or other collected variables. In addition, our statistical method using the Youden index equally weighted sensitivity and specificity, but perhaps one or the other should have been more heavily weighted. However, any increase in the sensitivity of the model would result in decreased specificity, and there is a potential downside to increasing the model's false positive rate and misallocating scarce social work or other resources away from the most at-risk patients. Because we identified pre-existing debt as a risk factor for financial toxicity in the model, it is possible that some of the identified financial toxicity events were unrelated to medical therapy and rather resulted from baseline debt. Thus, we cannot definitively say to what extent the model predicted financial toxicity resultant from RT in a vulnerable subpopulation versus simply identifying inevitable financial events in at-risk patients. However, the time course for the development of financial toxicity was most consistent with therapy-related economic stress, particularly in the context of literature documenting that patients with cancer are almost 3 times as likely to file for bankruptcy as are their peers who do not have cancer and that patients who are receiving therapy are more likely to file for bankruptcy than patients who are not.^4^ Lastly, this tool simply aids with identification of patients at risk for financial toxicity, but the ideal timing or type of intervention to mitigate toxicity is unknown.

To facilitate clinical implementation, we will soon publish a web-based or mobile-app integrated screening tool to provide a quick and easy method for clinicians to screen patients.

## Conclusions

Early identification of oncology patients at risk for financial toxicity is essential to improving patient quality of life and health outcomes, but validated general oncology screening tools are cumbersome and not specific to challenges related to radiation therapy. We retrospectively analyzed data from a prospective survey study conducted at a large referral center^11^ and propose a rapid, no-cost, sensitive, and specific financial toxicity screening tool that may minimize barriers to widespread clinical implementation by associating the incidence of patient-reported financial toxicity shortly after delivery of radiation therapy with answers to just 3 simple questions. Future work is necessary to validate this tool in an additional patient population and prospectively evaluate the best approach to mitigate treatment-related financial toxicity.

## Appendix Supplementary materials

Image, application 1

## Footnotes

Supplementary material associated with this article can be found in the online version at doi:10.1016/j.adro.2021.100782.
