# Supplementary material for: Development of a Financial Toxicity Screening Tool for Radiation Oncology: A Secondary Analysis of a Pilot Prospective Patient-Reported Outcomes Study
Source: Adv Radiat Oncol. 2021 Sep 11;6(6):100782. doi: 10.1016/j.adro.2021.100782 (PMC8503853; doi:10.1016/j.adro.2021.100782)
Supplement: Supplementary file 1 [file mmc1.docx]

Supplementary Table 1 Relationship between patient demographic characteristics and likelihood of developing of financial toxicity

|  |  | **Development of financial toxicity** | |  |
| --- | --- | --- | --- | --- |
| **Patient Demographics** | **Entire cohort (%)** | **No (%)** | **Yes (%)** | **P value*** |
| **Entire cohort (n= 157)** | 100.0 | 78.3 | 21.7 |  |
| **Gender (n=154)** |  |  |  | 0.44 |
| Male | 52.2 | 80.5 | 19.5 |  |
| Female | 45.9 | 75.0 | 25.0 |  |
| Transgender | 0.6 | 100.0 | 0.0 |  |
| **Race/ethnicity (n=156)** |  |  |  | 0.55 |
| Black or African-American | 17.8 | 71.4 | 28.6 |  |
| White/non-Hispanic | 70.7 | 80.2 | 19.8 |  |
| Other | 10.6 | 76.5 | 23.5 |  |
| **Age (n=156)** |  |  |  | <.01 |
| >80 | 5.8 | 100.0 | 0.0 |  |
| 71-80 | 14.6 | 91.3 | 8.7 |  |
| 61-70 | 43.9 | 84.1 | 15.9 |  |
| 51-60 | 22.3 | 65.9 | 37.1 |  |
| 20-50 | 12.6 | 60.0 | 40.0 |  |
| **Marital Status (n=150)** |  |  |  | 0.58 |
| Never married | 19.1 | 80.0 | 20.0 |  |
| Divorced/widowed/separated | 20.4 | 71.9 | 28.1 |  |
| Married | 56.1 | 80.7 | 19.3 |  |
| **Education (n=154)** |  |  |  | 0.20 |
| Did not complete high school | 9.6 | 86.7 | 13.3 |  |
| High school diploma | 48.4 | 81.6 | 18.4 |  |
| Associates degree | 8.9 | 57.1 | 42.9 |  |
| Undergraduate degree | 21.7 | 70.6 | 29.4 |  |
| Graduate degree | 9.6 | 86.7 | 13.3 |  |
| **Household Income (n=133)** |  |  |  | 0.45 |
| $84,000-185,000 | 19.7 | 87.1 | 12.9 |  |
| $53,000-84,000 | 17.8 | 67.9 | 32.1 |  |
| $31,000-53,000 | 20.4 | 84.4 | 15.6 |  |
| $12,000-31,000 | 18.5 | 75.9 | 24.1 |  |
| <$12,000 | 8.3 | 69.2 | 30.8 |  |
| **Debt (n=146)** |  |  |  | 0.11 |
| >$105,000 | 16.6 | 80.8 | 19.2 |  |
| $45,000-105,000 | 12.8 | 70.0 | 30.0 |  |
| $25,000-$45,000 | 9.6 | 66.7 | 33.3 |  |
| $5,000-$25,000 | 20.4 | 68.8 | 31.2 |  |
| <$5,000 | 33.8 | 84.9 | 15.1 |  |
| **Insurance Provider (n=152)** |  |  |  | 0.11 |
| Private | 47.1 | 73.0 | 27.0 |  |
| Public | 49.6 | 84.6 | 15.4 |  |
| **Understanding Insurance (n=156)** |  |  |  | 0.67 |
| Yes | 13.4 | 76.2 | 23.8 |  |
| No | 7.0 | 90.9 | 9.1 |  |
| Not sure | 79.0 | 77.4 | 22.6 |  |
| **Malignancy (n=153)** |  |  |  | <.01 |
| Breast | 22.9 | 72.2 | 27.8 |  |
| Central nervous system | 3.9 | 83.3 | 16.7 |  |
| Gastrointestinal | 3.9 | 71.4 | 28.6 |  |
| Genitourinary | 25.5 | 100.0 | 0.0 |  |
| Gynecologic | 2.6 | 100.0 | 0.0 |  |
| Gynecologic | 2.6 | 100.0 | 0.0 |  |
| Head and neck | 20.9 | 59.4 | 40.6 |  |
| Hematologic | 3.3 | 80.0 | 20.0 |  |
| Musculoskeletal and skin | 3.3 | 40.0 | 60.0 |  |
| Benign disease | 0.7 | 100.0 | 0.0 |  |

*Fisher’s exact test

Supplementary Table 2 Relationship between patient financial concerns and likelihood of developing of financial toxicity

|  |  | **Development of financial toxicity** | |  |
| --- | --- | --- | --- | --- |
| **Level of financial concern, by type** | **Entire Cohort (%)** | **No (%)** | **Yes (%)** | **P value*** |
| **Overall financial concern (n=154)** |  |  |  | <.01 |
| Very worried | 20.4 | 53.1 | 46.9 |  |
| Somewhat worried | 31.8 | 74.0 | 26.0 |  |
| Not worried | 45.9 | 91.7 | 8.3 |  |
| **Overall therapy costs (n=151)** |  |  |  | <.01 |
| Very worried | 7.0 | 81.8 | 18.2 |  |
| Somewhat worried | 26.1 | 51.2 | 48.8 |  |
| Not worried | 63.1 | 89.9 | 10.1 |  |
| **Copayments (n=154)** |  |  |  | <.01 |
| Very worried | 10.2 | 37.5 | 62.5 |  |
| Somewhat worried | 24.8 | 67.7 | 33.3 |  |
| Not worried | 63.1 | 89.9 | 10.1 |  |
| **Deductible (n=154)** |  |  |  | <.01 |
| Very worried | 12.1 | 47.4 | 52.6 |  |
| Somewhat worried | 26.1 | 73.2 | 26.8 |  |
| Not worried | 59.9 | 88.3 | 11.7 |  |
| **Concerned enough talk with provider (n=155)** |  |  |  | <.01 |
| Yes | 10.8 | 58.8 | 41.2 |  |
| Not sure | 14.6 | 69.6 | 30.4 |  |
| No | 73.2 | 83.5 | 16.5 |  |
| **Household costs (n=155)** |  |  |  | <.01 |
| Very worried | 11.5 | 61.1 | 38.9 |  |
| Somewhat worried | 24.8 | 56.4 | 43.6 |  |
| Not worried | 62.4 | 90.8 | 9.2 |  |

*Fisher’s exact test
